# Notes on Epidemic Scarlet Fever and Measles

**Published:** 1847-02

**Authors:** Silas Ames

**Affiliations:** Montgomery, Ala.


ART. III.—*Notes on Epidemic Scarlet Fever and Measles.* By SILAS AMES, M.D., of Montgomery, Ala.

These diseases prevailed in an epidemic form in Montgomery and its vicinity, during a part of the winter and spring of 1844. In their progress they presented some remarkable anomalies, which I propose to describe by some extracts from my note-book. The notes will be found to have but little practical value, but the facts recorded are interesting as opposing a theory of the simplicity of the diseases, which was advocated by Mr. Hunter, and which, having been assumed by Hahnemann as the basis of his therapeutics, his followers, many of them at least, believe to be necessary to the integrity of his system. The reader will observe that the observations extend somewhat beyond the peculiarities of these epidemics, though chiefly confined to them.

The scarlet fever made its appearance in January. The first case that I saw occurred on the 26th of that month; besides the symptoms common to the disease, this case was attended with acute bronchitis. After this time it spread very slowly until the latter part of April, when it declined rapidly, and disappeared finally in May, being most prevalent about the first of April.

The measles began in the latter part of March, were most prevalent in April, and declined in the latter part of May.

Each disease wore the livery of the other in a more marked manner than usual, but each maintained its distinctive features so far that there was no difficulty, in any well marked case, even when the two seemed blended, in determining which predominated. There were two cases in which this difficulty occurred, but the symptoms in both were very mild and the rash imperfectly developed. Before the measles appeared, cough was a frequent symptom of scarlet fever, but afterwards became much more common; and catarrhal symptoms frequently preceded the eruption in the latter. On the other hand, the measles was generally attended with more decided anginose symptoms than common, and occasionally they were of very great severity. In two instances the angina of measles terminated in acute laryngitis, and in one other in croup. So great indeed was the resemblance which the symptoms of the eruptive fever of the one bore to those of the other, that I had occasionally to alter the name on my register after the eruption appeared.

During the continuance of these two diseases there prevailed also remittent and intermittent fevers, diarrhœa and bronchitis, to such an extent that, taking into consideration the number, and the time of the year, either might deserve to rank as an epidemic. I did not observe, however, that the fevers differed from our summer endemic fevers either in their symptoms, or in the effect of the usual treatment; nor did I observe that any other disease prevailing at the same time partook of the characters of scarlet fever or measles; but the latter were greatly influenced by the cause of the remittent and intermittent fevers. The whooping cough, which also prevailed to some extent, was conjoined in several instances, in the same person, with scarlet fever and measles, but could not be said to partake of the nature of either, though the former was somewhat modified by the conjunction, and its danger greatly aggravated. The whooping cough did not disappear with the other diseases, but lingered on, with scarcely a perceptible existence, until the spring of the following year, when it spread more generally. There were also sporadic cases of pneumonia, pleuritis, and dysentery.

The weather which preceded this unusual assemblage of diseases deserves some notice. Although the quantity of rain which fell was not much greater than common, the fall and winter were remarkable for the number of cloudy and wet days; so much so that the roads got into a worse condition than had been known before, and the untrodden land became miry and unsafe. This kind of weather continued from early in November until about the tenth of February, when it was suddenly displaced by the opening of spring, early as was the season, in more than its usual mildness. Nearly six weeks of uninterrupted dry and warm weather succeeded.

Scarlatina. *Varieties.* Generally anginose.—I saw but few cases of the malignant, and but one of the simple form.

*Eruptive fevers.*—This was generally remittent, but often continued, in a few cases intermittent, and in a few others congestive. The forming stage was always short, and sometimes there were no premonitory symptoms.

Case I.—G., two years old, was attacked on the 12th of February. He was taken suddenly, while in apparent good health, with a slight chill, followed by moderate fever, hoarseness, and sore throat. On the 14th, he had the usual symptoms of scarlet fever, with cough, except that the type of the fever was that of a regular quotidian intermittent. The exacerbation began about four o'clock in the afternoon, and declined from twelve at night until morning, when the intermission was complete. On the eighth day of the disease the fever assumed a continued form, and the affection of the throat increased in severity, accompanied by urgent symptoms of laryngitis. He died of the latter affection on the next day.

Case II.—Sarah, a young mulatto woman, was attacked suddenly on the 12th of March. I visited her twelve hours afterwards; at which time the skin on the head and trunk was cool, and cold on the extremities; pulse 150, barely distinct enough to be counted; respiration very quick; delirium; stupor; cough; palate and tonsils much swollen; great restlessness; whispers hoarsely in speaking; no eruption. She died within twenty-four hours from the beginning of the attack.

Case III.—Louisa, aged three years, daughter of Sarah, was attacked on the same day, the 12th. Symptoms the same as her mother's, except that she had no sore throat, and the stupor was succeeded by convulsions. She died within thirty hours from the beginning of the attack.

*Angina.*—The principal peculiarity in the affections of the throat was the tendency to laryngeal inflammation. This occurred very often, and was the cause of death in many of the fatal cases. The inflammation sometimes covered the pharynx, as far as it was visible, in every direction. The white patches on the fauces did not always cover an ulcer. In several cases I saw dark and fœtid ulcers on the tonsils, and in one on the posterior palatine arch, without other malignant symptoms.

*Eruption.*—The eruption usually appeared on the second day, but frequently on the third. In a few cases it partook of the character of that of measles: in one case it alternated with it; and in another both eruptions were out distinctly at the same time on different parts of the skin.

Case IV.—B. P., aged eight months, having had whooping cough ten or twelve days, this disease being fully formed, was attacked with scarlet fever on the 18th of April. On the first and second days afterwards the cough was mitigated, but on the third day, the eruption having come out freely, the cough resumed its former violence with whooping. On the fifth and sixth days the eruption resembled that of measles in several particulars. It had taken a darker hue, was in patches, separated by portions of clear skin, contrasting strongly with the affected parts, and was elevated; but the patches were neither so small as in measles nor so circular or crescentic in form. On the seventh day, the eruption remaining unchanged, he had symptoms of hydrocephalus; on the eighth convulsions; and died on the ninth comatose.

Case V.—H. W., five years old, was attacked on the 15th of April with the usual symptoms of scarlet fever, and cough. On the third day the eruption was very distinct and had the characteristics of this disease. On the fourth day, in the morning, these characteristics had disappeared, and those of measles had taken their place; at the same time his face had become tumid, and his eyes red, suffused, and painful in the usual light. The eruption had now the distinctive signs of measles,—in its appearance on the fourth day, in being accompanied by catarrhal symptoms, in occurring in small patches, circular or crescent shaped, elevated and dark, with intervening portions of skin not affected. On the fifth day, the symptoms of measles were mitigated or absent; the rash though faded had resumed the appearance of scarlet fever, except on a small part of the arms near the wrists, and on the knees, where it had a vivid color, and retained the appearance of measles. On the sixth day, no trace of measles could be discerned except the cough, which was greatly aggravated. The ulcers on the tonsils had become deeper, dark, and offensive; diarrhœa had supervened; the abdomen had become tumid and tender, and the tongue dry and fissured. He died on the seventh day, apparently from enteritis.

Case VI.—L. W., sister to H. W., aged ten years, was attacked on the 23d of April. The symptoms were those common to scarlatina, except an almost continued sweating, which at that time was a common symptom of measles, and that the fever was a quotidian intermittent, distinct and regular, and accompanied by a slight cough. The eruption came out on the third day, and occupied more surface than was common. On the fifth day, a catarrh, as decided as in any case of measles, appeared with an increase of cough. At the same time there was such a marked change in the appearance of the eruption as to be noticed with great alarm by her mother, who asked me as soon as I entered the room if the disease was changing to measles. Her attention was confined to the eruption, but on examining it carefully I could see no change except in the color: it was darker, and had more of the raspberry tint of measles. On the seventh day, the catarrh continuing, the eruption had disappeared entirely from the face and neck, and was faded everywhere else. On the eighth day of the disease, and fourth of the catarrh, the eruption of measles showed itself very plainly on the face, neck, shoulders, and upper part of the chest. On the ninth day, it was fully developed, without being extended beyond the parts which it occupied the day before. I did not observe any change in the scarlet rash; it did not recede, but remained in the same state as on the seventh day, until the rash of the measles began to fade, and then followed the course of the latter, so that desquamation did not begin before the thirteenth day. After the measly rash came out the fever became continued, without any abatement of the perspiration. The whole course of the complaint was mild, and convalescence went on favorably.

*Sine eruptione.*—There were seventeen cases of this kind, eight of which occurred in adults; six of the whole number, I was informed, had had scarlet fever; two others I saw with it in 1840, and one other in 1833; neither of the latter was adult.

*Complications.*—Besides the complications with measles, there were four cases conjoined with whooping cough. Two of these proved fatal. In all of them the cough preceded the fever.

*Crisis.*—If favorable, generally on the fifth or seventh day; if unfavorable, generally on the ninth. I did not notice any critical discharges.

*Sequelæ.*—Generally anasarca. I observed in one case an obstinate urticaria during convalescence, and in several cases aphthæ.

*Treatment.*—I refer to the treatment chiefly for the purpose of making a single observation, viz: that the remedies, or remedy, which are most successful in the treatment of scarlet fever in the epidemic of one year cannot be depended on, as a general rule, in that of another year. The truth of this observation is admitted by the profession, I believe, in regard to epidemics generally, but there is supposed to be a character of specialty about what are termed specific or contagious diseases, particularly the exanthemata, which secures a uniform result, or an approach to it, from the same treatment whenever they appear. Although the records of medicine afford abundant evidence that this is an error, there are, nevertheless, but few of even our best systematic authors who do not aid in confirming it, by recommending in this class of fevers, a general plan of treatment, depleting or stimulant; frequently giving prominence to a particular remedy of great power, which is intended to be applied at all times when the same assemblage of symptoms is to be combated. My own experience leads to a practical conclusion the reverse of this, and in accordance with the observation made above.

In regard to scarlet fever, this is the third epidemic of the kind I have seen. The first, which preceded the cholera here, began in May, 1833. In this bleeding, general or local, was the most efficient remedy, and, indeed, almost the only one required. Blood could be drawn safely, and generally with the greatest benefit, in either variety, and in any stage of the disease. In the second, which took place in the spring of 1840, being influenced by my knowledge of its benefit in the first, I again resorted to bloodletting, and recommended it to others, with the greatest confidence; but unfortunately with opposite results. It was decidedly and speedily injurious in every instance, apparently rendering mild cases bad ones, and bad ones fatal. There was a tendency to the formation of abscesses about the throat externally; and I observed that after bleeding, the tendency was increased so far that large ones frequently gathered about the puncture of the lancet or scarificator. When local inflammation existed, which was not uncommon, the loss of blood seemed rather to aggravate than to relieve it, and I am confident, notwithstanding I express the opinion with much hesitation, that in the absence of any local inflammation, bleeding produced it. I do not think I could be mistaken in this opinion, for I had a large opportunity for observation, and the conclusion was forced on me against all that I had seen of the disease, to say nothing of the teachings and prejudices of medical books. A peculiarity in the symptoms led me to the use of cathartics, which proved to be quite as beneficial in this, as bleeding had been in the former epidemic; the result being equally favorable when the tongue was clean, red, and dry, and the evacuations watery. The latter, however, was rarely the case, as in most instances there was an unaccountable accumulation of feces from day to day.* In the third, that of 1844, I derived very little benefit from any constitutional remedies. I varied them sufficiently, and continued them long enough to become satisfied, that neither the bad nor the mild cases were at all influenced by them as *curative* agents. As palliatives, sponging the body with warm or cold water, and, in the congestive variety, hot salt-water baths, and mustard emetics, were of service. In contrast with the treatment in 1833 and 1840, evacuations from the bloodvessels or bowels were never advantageous in this, even as paliatives. I obtained, however, the most decided advantage from the use of a strong solution of nitrate of silver applied to the palate and tonsils; so much so, that seeing so little good effect from other remedies, and becoming satisfied that the greatest danger consisted in an extension of the inflammation of the fauces to the larynx, I came, after a time, to treat the disease almost solely as a local affection. I must add, however, in concluding the subject, that I have

---

* Sir Gilbert Blane records a similar fact, in a case of chorea, "in which the vitiated quality, incredible quantity, and long continuance of alvine *sordes*, was such as to bid defiance to all the principles of physiology and pathology to account for."—*El. Med. Logic, sec. IV*

been informed, on authority that does not permit me to doubt the fact, that Dr. Guild treated the scarlet fever which prevailed in Tuscaloosa at the same time, very successfully, with colchicum, a remedy which I did not use.

Measles. *Eruptive fever.*—Generally remittent; frequently intermittent; rarely continued. In a few instances the catarrhal symptoms did not appear before the eruption; sweating was common in this as well as in the stage of eruption. Vomiting was also a common symptom, ushering in the disease as in scarlet fever.

Case I.—W. P., three years old, was taken with measles on the 25th of April. Fever, a tertian intermittent, with continued perspiration except at the very beginning of the exacerbation; the cold stage, if any, was not noticed. On the fourth day, when the eruption came out, the fever remitted only, and afterwards became continued. On the ninth day, the measles having finished its course in the usual way, there was an intermission; on the tenth day, an exacerbation of fever, preceded by a slight chill. On the eleventh day, an intermission; and on the twelfth, another exacerbation. He took quinine on the next day and night, and had no return of fever.

Case II.—J. H., aged five years, was attacked on the 28th of April. Fever, a quotidian intermittent, accompanied with profuse sweating. The fever continued to have this form, with the usual symptoms of measles in other respects, until the fourth day, when the eruption appeared, and the fever remitted only; afterwards there was no remission till the disease terminated.

I saw several other cases of this kind; the fever, previously intermittent, remitting on the day of the first show of the rash and then becoming continued as soon as the rash had come out fully.

I have neglected to state, in the proper place, that the appearance of the rash in scarlatina did not perceptibly change the type of the fever. The fact, however, is exemplified in case 1.

*Angina.*—This was of extraordinary severity in a majority of the cases which occurred previously to the first of May. There was not often white patches, nor ulceration of the throat. I have noted but four cases, two of which had laryngitis. One of the latter proved fatal; the laryngeal affection coming on on the same day, and terminating on the ninth. In one other case the inflammation attacked the trachea, producing the ordinary symptoms of croup. In this case the throat was not ulcerated.

*Eruption.*—In most cases the papillæ were elevated on the extremities, and the itching of the skin was very annoying. In two cases the eruption resembled that of scarlet fever.

Case III.—A. P., aged eighteen months, was attacked with measles, after having had scarlet fever, before convalescence was established, and whilst the skin was still discolored by its rash. She had great soreness of the throat, with swelling of the tonsils, on which could be seen two or three aphthous patches, and her tongue was like the tongue in scarlet fever. She had diarrhœa also, which was then a common symptom of measles. The eruption bore a greater resemblance to scarlet fever than to measles.

Case IV.—Mrs. E., was taken with a chill on the 4th of June; had a quotidian intermittent fever until the 7th, when it changed to a continued type. She had from the first catarrhal bronchitis, and also some soreness of the throat.—On the 9th an eruption came out on the face, breast, and arms, which spread during the day and night over the rest of the body, including the lower extremities. I visited her on the 10th; the eruption had all the characters of scarlatina (levigata) with a very troublesome itching of the skin. When examined from a little distance, there were perceptible on the face and neck, but not elsewhere, through the otherwise uniform scarlet blush, a number of darker spots, not elevated, representing very accurately the flea-bite appearance of the eruption of measles.

The following table will show the number of cases of scarlet fever and measles in my own practice, and of other diseases that prevailed at the same time, with their relative mortality:

| | No. of cases. | deaths |
|---|---|---|
| Scarlet fever,.................................... | 64 | 5 |
| Scarlet fever conjoined with whooping cough,............ | 4 | 2 |
| Measles,.......................................... | 30 | 2 |
| Measles, with whooping cough,.......................... | 6 | 4 |
| Whooping cough,..................................... | 16 | .. |
| Remittent and intermittent fevers,...................... | 78 | .. |
| Diarrhœa and dysentery,............................... | 50 | 1 |
| Bronchitis, pneumonia, and pleuritis,.................. | 46 | 1 |

Montgomery, Dec. 22, 1846.

